# Staphylococcal β-Toxin Modulates Human Aortic Endothelial Cell and Platelet Function through Sphingomyelinase and Biofilm Ligase Activities

**DOI:** 10.1128/mBio.00273-17

**Published:** 2017-03-21

**Authors:** Alfa Herrera, Katarina Kulhankova, Vijay K. Sonkar, Sanjana Dayal, Aloysius J. Klingelhutz, Wilmara Salgado-Pabón, Patrick M. Schlievert

**Affiliations:** a Department of Microbiology, University of Iowa Carver College of Medicine, Iowa City, Iowa, USA; b Department of Internal Medicine, University of Iowa Carver College of Medicine, Iowa City, Iowa, USA; University of Wisconsin

**Keywords:** beta-toxin, biofilm ligase, sphingomyelinase, *Staphylococcus aureus*, endothelial cells

## Abstract

*Staphylococcus aureus* causes many infections, such as skin and soft tissue, pneumonia, osteomyelitis, and infective endocarditis (IE). IE is an endovascular infection of native and prosthetic valves and the lining of the heart; it is characterized by the formation of cauliflower-like “vegetations” composed of fibrin, platelets, other host factors, bacteria, and bacterial products. β-Toxin is an *S. aureus* virulence factor that contributes to the microorganism’s ability to cause IE. This cytolysin has two enzymatic activities: sphingomyelinase (SMase) and biofilm ligase. Although both activities have functions in a rabbit model of IE, the mechanism(s) by which β-toxin directly affects human cells and is involved in the infectious process has not been elucidated. Here, we compared the *in vitro* effects of purified recombinant wild-type β-toxin, SMase-deficient β-toxin (H289N), and biofilm ligase-deficient β-toxin (H162A and/or D163A) on human aortic endothelial cells (HAECs) and platelets. β-Toxin was cytotoxic to HAECs and inhibited the production of interleukin 8 (IL-8) from these cells by both SMase and biofilm ligase activities. β-Toxin altered HAEC surface expression of CD40 and vascular cell adhesion molecule 1 (VCAM-1). HAECs treated with β-toxin displayed granular membrane morphology not seen in treatment with the SMase-deficient mutant. The altered morphology resulted in two possibly separable activities, cell rounding and redistribution of cell membranes into granules, which were not the result of endosome production from the Golgi apparatus or lysosomes. β-Toxin directly aggregated rabbit platelets via SMase activity.

## IMPORTANCE

Each year there are up to 100,000 cases of infective endocarditis (IE) in the United States. *S. aureus* is the most common pathogen in patients with health care-associated IE and the leading cause of community-associated IE in the developed world. Multiple clonal group strains as defined by the Centers for Disease Control and Prevention, particularly USA200 and other clones encoding β-toxin, are highly associated with IE. Considering the strong association and established contribution of β-toxin in animal models of IE, determining how β-toxin directly affects human cell types, including endothelial cells and platelets, is important. In this study, we demonstrate that β-toxin functions to modulate endothelial cells and platelets by both toxin sphingomyelinase and biofilm ligase activities. Our data suggest that these activities modulate inflammation and increase infection severity.

## INTRODUCTION

*Staphylococcus aureus* is a Gram-positive bacterium that causes various minor infections, including superficial skin and soft tissue infections, and life-threatening infections, such as toxic shock syndrome, pneumonia, osteomyelitis, sepsis, and infective endocarditis (IE) (1–9). IE is an endovascular infection of native and prosthetic valves and the lining of the heart usually caused by Gram-positive cocci. The infection is characterized by the formation of “vegetations,” which are cauliflower-like structures composed of fibrin, platelets, inflammatory cells, and other host factors, along with aggregated bacteria and their products. There are up to 100,000 cases of IE in the United States yearly (10). *S. aureus* is the most commonly identified pathogen in patients with health care-associated IE, accounting for 40% of cases, and the leading cause of community-associated IE in the developed world (9). Furthermore, *S. aureus* is the second most commonly identified pathogen from bloodstream infections, which is a major risk factor for developing IE (11). Once patients contract *S. aureus* IE, various complications, such as pulmonary infarctions, strokes, and congestive heart failure, occur. The IE complications make this infection more lethal, resulting in hospital-associated deaths in up to 65% of cases (9, 10, 12). Additionally, 30 to 40% of the population is colonized with *S. aureus*, which increases the possibility that the bacteria will reach the bloodstream to cause IE.

Damage to the heart and valves from congenital heart defects, rheumatic valve disease, diabetes, immunosuppression, or advancing age increases the odds of developing *S. aureus* IE (13). Furthermore, the risk of developing IE caused by *S. aureus* is greatly increased because of the rise in prosthetic device implantation and surgical procedures (13). While *S. aureus* readily attaches to damaged valves, the microorganism does not require prior vascular damage for adherence.

Endothelial cells line heart valve surfaces, and not only do they serve to maintain structural integrity and act as barriers but they also function in regulating the immune system (14). These cells are affected in many diseases related to endothelial injury, dysfunction, and activation and influence atherosclerosis, hypertension, sepsis, and inflammatory syndromes (14). When damage occurs to endothelial valve surfaces, their structure is altered significantly. This in turn leads to altered blood flow, and sterile vegetations can form due to platelet and fibrin deposition and deposition of other host factors, recruited to repair damage. This deposition process inadvertently promotes *S. aureus* colonization, and thus IE vegetation formation. After colonization, *S. aureus* virulence factors contribute to host cell damage, thereby continuing to promote vegetation formation; this occurs by increasing platelet and fibrin deposition, as well as amplifying injury through recruitment of immune cells and modification of the inflammatory state (15).

Platelets are critical in the development of IE due to involvement in wound healing and as components of the innate immune system. The recruitment of platelets and their aggregation augments bacterial colonization of valves, as many *S. aureus* adhesion factors bind to platelets. Furthermore, platelet activation results in secretion of more than 300 proteins from granules that recruit other immune cells and more platelets, reduce vasoconstriction, increase inflammation, and have antimicrobial activity (16). Some bacteria also actively recruit platelets into infection sites and activate the platelets, resulting in the formation of septic thrombi, such as those formed during IE. Bacteria become embedded in the thrombi and are protected from the host immune system, supporting bacterial persistence (16).

A prevalent *S. aureus* virulence factor is the cytolysin β-toxin. The toxin is a member of the DNase I superfamily and is 35 kDa in size, folding into a four-layer sandwich with two β-sheets at the center (17, 18). β-Toxin is characterized by its ability to lyse red blood cells as the hot-cold hemolysin and kill immune cells by its neutral sphingomyelinase (SMase) activity that digests sphingomyelin into ceramide and phosphocholine. β-Toxin also has a second less characterized biofilm ligase activity involved in promoting biofilm formation (17, 18).

β-Toxin is important in IE development. Vegetation formation and lethality are both increased when β-toxin is produced by infecting strains in a rabbit model of IE and sepsis (19, 20). Both SMase and biofilm ligase contribute to increasing vegetation size (20). However, the SMase activity also appears to increase lethality (20).

While β-toxin is important for IE, how the toxin interacts with host cells directly at infection sites has not been studied. We therefore examined β-toxin host cell effects in the development of IE by studying the effects that β-toxin and its two known properties have on human aortic endothelial cells (HAECs) and platelets. β-Toxin is cytotoxic to HAECs and inhibits the production of interleukin 8 (IL-8) from these cells by its SMase and biofilm ligase activities. β-Toxin alters HAEC surface expression of CD40 and vascular cell adhesion molecule 1 (VCAM-1). We noted HAEC rounding when treated with wild-type and SMase- and biofilm ligase-deficient mutants. When stained with wheat germ agglutinin (WGA), HAECs treated with β-toxin displayed granular cell morphology not seen in treatment with the SMase-deficient mutant. Last, we determined that β-toxin directly activates platelets.

## RESULTS

### SMase and biofilm ligase activity reduce β-toxin HAEC cytotoxicity.

**FIG 1:**
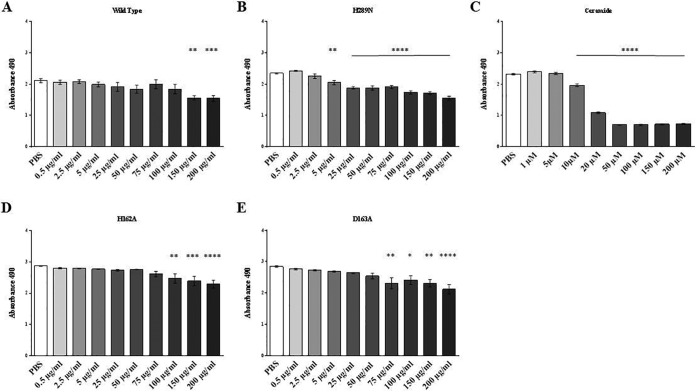
β-Toxin is cytotoxic to HAECs in a dose-dependent manner. (A to E) MTS cytotoxicity assays were performed with wild-type β-toxin (A), SMase-deficient mutant H289N (B), ceramide (C), and biofilm ligase-deficient mutants H162A (D) and D163A (E). Asterisks denote significance in relation to PBS-treated cells. Absorbance at 490 nm is shown on the *y* axes. *, *P* < 0.05; **, *P* < 0.01; ***, *P* < 0.001; ****, *P* < 0.0001.

To begin to assess the effects of β-toxin on HAECs and by which mechanisms of action, we completed 3-(4,5-dimethylthiazol-2-yl)-5-(3-carboxymethoxyphenyl)-2-(4-sulfophenyl)-2H-tetrazolium (MTS) cytotoxicity assays for HAECs treated individually with purified recombinant wild-type β-toxin, SMase-deficient β-toxin (H289N), and biofilm ligase-deficient β-toxin (H162A and D163A) (17, 20). Cytotoxicity was assessed after treatment with β-toxin concentrations from 0.5 to 200 µg/ml (~15 to 6,000 nM) compared to cells mock treated with phosphate-buffered saline (PBS). It is important to note that β-toxin concentrations within *in vitro* biofilms may exceed 15,000 µg/ml (P. M. Schlievert, unpublished data), thus indicating that our chosen concentrations are likely to be physiological. Wild-type β-toxin was cytotoxic to HAECs only at the highest concentrations tested, 150  µg/ml (4,500 nM) and 200 µg/ml (6,000 nM) (Fig. 1A). Similarly, the biofilm ligase mutants (D163A and H162A) were cytotoxic to HAECs at high concentrations (≥75 µg/ml is equivalent to ≥2,200 nM) (Fig. 1D and E). However, the SMase-deficient mutant, the H289N mutant, was cytotoxic to the cells at significantly lower concentrations, beginning at 5 µg/ml (150 nM) (Fig. 1B). Ceramide, which is produced as a result of the SMase activity cleaving sphingomyelin, was cytotoxic to the HAECs at 10,000 nM (10 µM) (Fig. 1C).

### HAEC IL-8 production is inhibited by β-toxin through SMase and biofilm ligase activity.

**FIG 2:**
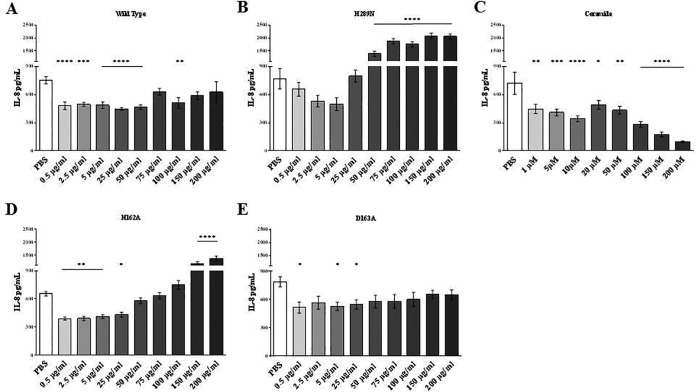
SMase and biofilm ligase activities of β-toxin inhibit the ability to induce production of IL-8 by HAECs. (A to E) ELISAs for IL-8 were performed on supernatants from HAECs treated with wild-type β-toxin (A), β-toxin lacking SMase activity (B), ceramide (C), and β-toxin mutants lacking biofilm ligase activity (D and E). Asterisks denote significance in relation to PBS-treated cells. *, *P* < 0.05; **, *P* < 0.01; ***, *P* < 0.001; ****, *P* < 0.0001.

Proinflammatory states facilitate endothelial damage and immune dysfunction during IE. Previous data demonstrate that β-toxin inhibits IL-8 production in human umbilical vein endothelial cells (HUVECs), and therefore, we investigated the ability of β-toxin and mutants to inhibit IL-8 production in HAECs (21). Wild-type β-toxin inhibited IL-8 production in HAECs at toxin concentrations from 0.5 to 50 µg/ml (15 to 1,500 nM), but when the toxin concentration was more than 100 µg/ml, there was no significant inhibition of IL-8 production (Fig. 2A). The two biofilm ligase mutants (D163A and H162A) also inhibited IL-8 production, but at concentrations between 0.5 and 25 µg/ml (15 to 750 nM) (Fig. 2D and E). The H162A mutant exhibited markedly upregulated production of IL-8 in the endothelial cells at ≥150 µg/ml (≥1,500 nM), stimulating production significantly higher than for PBS treatment. IL-8 production was not inhibited at any concentration tested by the SMase mutant (Fig. 2B). Correspondingly, treatment with ceramide alone inhibited IL-8 production, including the lowest concentration tested of 1,000 nM (Fig. 2C).

### β-Toxin alters surface expression of CD40 and VCAM-1, but not ICAM-1, on HAECs.

**FIG 3:**
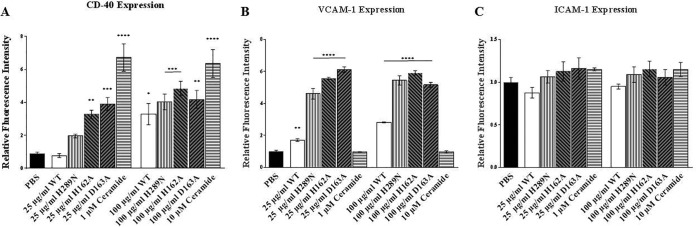
β-Toxin changes HAEC expression of CD40 and VCAM-1. In-cell Western immunoblotting was performed with HAECs. (A to C) Changes in CD40 expression (A), VCAM-1 expression (B), and ICAM-1 expression (C) from PBS-treated cells after treatment with wild-type β-toxin, SMase-deficient mutant, biofilm ligase-deficient mutants, and ceramide. Asterisks denote significance in relation to PBS-treated cells. *, *P* < 0.05; **, *P* < 0.01; ***, *P* < 0.001; ****, *P* < 0.0001.

HAEC surface regulatory molecules link innate and adaptive immunity. To determine whether β-toxin alters surface immune markers, we examined the expression of CD40, intercellular adhesion molecule 1 (ICAM-1), and vascular cell adhesion molecule 1 (VCAM-1). At 25 µg/ml (750 nM), wild-type β-toxin and SMase mutant (H289N) did not alter expression of CD40. However, the H162A and D163A biofilm ligase mutants upregulated expression of CD40 compared to PBS-treated cells (Fig. 3A). Ceramide at 1,000 nM caused a significant increase in CD40 expression. At 100 µg/ml (3,000 nM), wild-type β-toxin upregulated CD40 expression compared to PBS, as did both SMase and biofilm ligase mutants (Fig. 3A). Ceramide at 10,000 nM increased CD40 expression at levels comparable to treatment with 1,000 nM. When we examined VCAM-1 expression, wild-type β-toxin (25 µg/ml [750 nM]) increased levels of expression compared to PBS-treated cells (Fig. 3B). VCAM-1 expression was more significantly upregulated by both SMase and biofilm ligase mutants at the same concentrations (Fig. 3B). When the cells were treated with 100 µg/ml (3,000 nM) of wild-type β-toxin, VCAM-1 expression was significantly increased compared to PBS, but treatment with either type of mutant upregulated expression at even higher levels than wild-type β-toxin (Fig. 3B). When tested with ceramide, VCAM-1 levels were not altered in comparison to PBS at either 1,000 or 10,000 nM. None of the β-toxin protein toxins, wild type or mutants, or ceramide at either concentration affected ICAM-1 expression levels (Fig. 3C).

### HAEC membrane is disrupted by β-toxin SMase activity.

**FIG 4:**
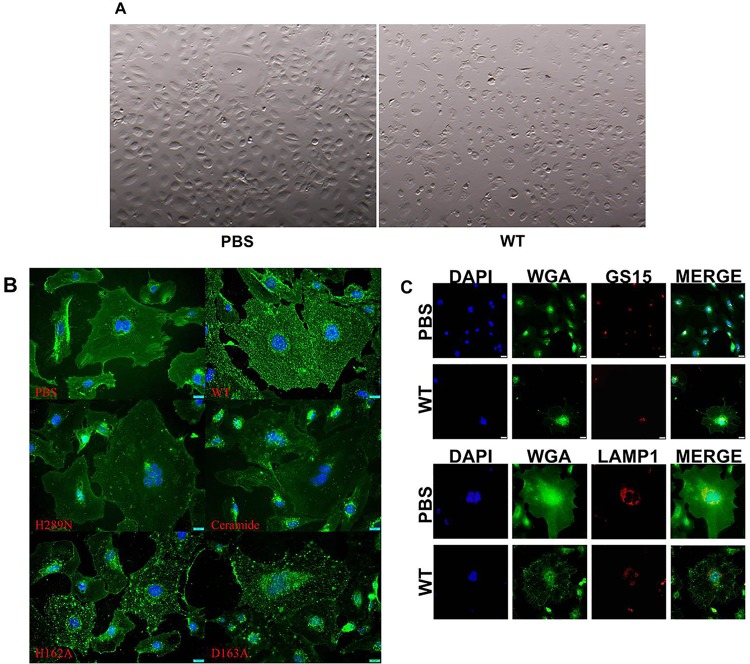
β-Toxin causes membrane alterations of HAECs. Images of HAECs under a dissecting microscope after treatment with β-toxin for 24 h are shown. (A) PBS-treated cells showing no change in cell morphology and retaining a cobblestone shape and HAECs treated with wild-type (WT) β-toxin, mutants, and ceramide-inducing cell rounding. (B) HAECs treated with β-toxin displaying granular distribution of wheat germ agglutinin (WGA) staining, PBS-treated cells show smooth even WGA staining throughout the cells, HAECs treated with wild-type or biofilm ligase mutants showing altered (speckled) cell membrane staining, treatment with the SMase mutant or ceramide appearing not to alter WGA cell membrane staining. (C) β-Toxin-induced speckling of HAECs resulting in the change to cell membrane morphology not due to the formation of endosomes from the Golgi apparatus or lysosomes. The top panel shows WGA does not colocalize with GS15 in β-toxin-treated cells differently from PBS-treated control HAECs. The bottom panel shows that LAMP-1 does not colocalize with WGA speckles formed from β-toxin treatment in HAECs.

β-Toxin causes lysis of human red blood cells and kills immune cells, and thus alters plasma membrane integrity. To begin to determine how β-toxin affects the HAEC membrane, we examined changes in cell morphology through optical and immunofluorescence microscopy. When we examined HAECs treated with wild-type β-toxin for 24 h through a dissecting microscope, we observed cell rounding at 150 µg/ml (4,500 nM), whereas the morphology of mock PBS-treated cells did not change (Fig. 4A). Cell rounding can be seen at the same concentration when treated with either of the biofilm ligase mutants (H162A and D163A). However, when cells were treated with the SMase mutant (H289N), cell rounding was observed at lower concentrations, beginning at 5 µg/ml (150 nM). Ceramide also caused cell rounding, beginning at 10,000 nM. To examine HAEC cell membrane structure, we stained the cells with wheat germ agglutinin (WGA) which binds to sialic acid and *N*-acetylglucosaminyl residues found on cell surface membranes. PBS-treated cells stained with WGA displayed even, smooth membranes (Fig. 4B). In contrast, wild-type β-toxin-treated HAECs had granular membrane staining throughout the cells (Fig. 4B). Similarly, the DNA biofilm ligase mutant-treated HAECs (H162A and D163A) also had granular appearances (Fig. 4B). When cells were treated with the SMase mutant (H289N), they did not have granules, but their appearance was similar to that of PBS-treated cells (Fig. 4B). Treatment with 5,000 nM ceramide did not cause granular appearance.

To determine whether the granules induced by β-toxin and biofilm ligase mutants were vesicles formed as a result of endoplasmic reticulum-Golgi protein transport or lysosome formation, we stained the cells for GS15 (*G*olgi *s*oluble N-ethylmaleimide-sensitive factor protein receptor with a size of *15* kDa) and lysosome-associated membrane glycoprotein 1 (LAMP1), markers for Golgi apparatus and lysosomes, respectively. While GS15 colocalized with WGA around the nucleus in β-toxin-treated cells, the same was observed with PBS-treated cells (Fig. 4C). LAMP-1 colocalized with WGA in PBS-treated cells as noted by the yellow merge color but did so much less in the β-toxin-treated cells (Fig. 4C).

### β-Toxin induces platelet aggregation.

**FIG 5:**
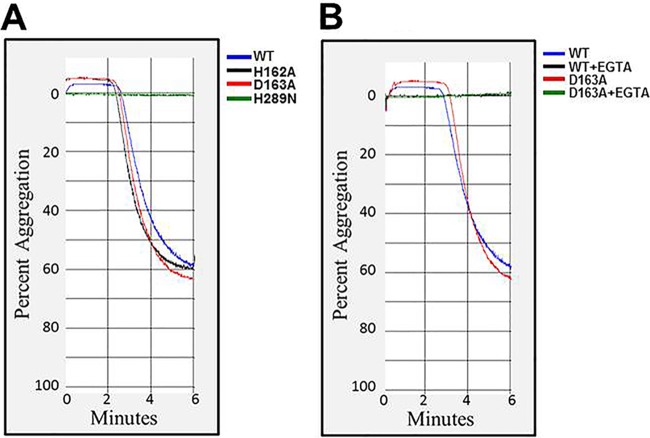
SMase activity of β-toxin induces platelet aggregation through integrin activation. (A) Representative aggregation curves showing treatment of platelets with wild-type β-toxin or H162A, D163A, and H289N mutants. H162A and H165A mutants induce robust aggregation response. However, treatment with the H289N mutant does not induce aggregation. (B) Pretreatment with EGTA inhibits the aggregation response invoked by wild-type β-toxin or the D163A mutant.

Platelets are important during IE, as they are recruited by the host to repair damage at the endothelium and are part of the innate immune system, affecting immune cell recruitment and inflammation. We hypothesized that β-toxin also activates platelets. Considering platelet aggregation as a functional evaluation of platelet activation, we performed platelet aggregation assays to determine whether β-toxin induces platelet activation. In control experiments, we first confirmed that treatment of washed rabbit platelets with collagen (a physiological platelet agonist), induced 65% ± 2% aggregation response (data not shown). Interestingly, treatment of rabbit platelets with wild-type purified β-toxin induced a similar robust response (60.3% ± 0.8% aggregation [Fig. 5A]). Treatment with both DNA biofilm ligase mutants (H162A or D163A) also induced similar levels of platelet aggregation (63% ± 1.5% and 66.3% ± 1.15% aggregation [Fig. 5A]). However, when we treated the platelets with the SMase mutant, the protein did not induce aggregation (Fig. 5A), suggesting that SMase activity, but not the biofilm ligase activity of β-toxin, is required to induce platelet aggregation.

Since platelet aggregation is linked with calcium-dependent activation of α_2b_β_3_ integrins on platelet surfaces, we performed control experiments to ensure that the observed aggregation responses represented activation of platelet integrins, rather than nonspecific platelet agglutination. We preincubated platelets with EGTA (5 mM) prior to β-toxin exposure with the premise that EGTA would chelate calcium and would inhibit aggregation but not prevent agglutination. When wild-type or D163A β-toxin was added to platelets pretreated with EGTA, platelet aggregation was completely inhibited (Fig. 5B), indicating that the aggregation of rabbit platelets with these toxins was due to integrin-dependent platelet activation.

## DISCUSSION

*S. aureus* IE is an acute infection affecting multiple host cell types. Several common risk factors increase the likelihood a person will contract IE infections, such as age, congenital heart defects, and artificial heart valve replacement, due to the damage they cause to the heart and blood vessels (13). These risk factors promote cell damage and thus vegetation formation. β-Toxin contributes to IE development and specifically increases vegetation formation, which in turn is predictive of clinical outcome and severity of disease (18–20).

In this study, we characterized how β-toxin, by its two mechanisms of action, impacts IE by examining its effects on HAECs. Our data show that wild-type β-toxin and biofilm ligase-deficient mutants are cytotoxic to HAECs, while the SMase-deficient mutant is even more toxic. β-Toxin inhibits the production of IL-8 from HAECs, and it represses this production via both of its mechanisms of action. The biofilm ligase activity regulates the amount of CD40 expression. Similarly, SMase and biofilm ligase activities limit β-toxin upregulation of VCAM-1 expression; ICAM-1 expression is unaffected by treatment with wild-type β-toxin or mutants.

Regulation of inflammation is necessary for the progression of IE and vegetation formation. Our data suggest that β-toxin functions to limit proinflammatory states via both mechanisms of action to increase the severity of disease.

To determine how β-toxin and its two activities contribute to local tissue effects in IE, we tested the cytotoxicity of purified wild-type and mutant toxin on HAECs *in vitro*. We used a broad range of β-toxin concentrations to represent the levels of β-toxin that may be produced by *S. aureus* strains. We observed that both wild-type toxin and biofilm ligase-deficient mutants caused cytotoxicity to HAECs in dose-responsive manners. Treatment with the mutant lacking SMase activity resulted in greater toxicity. These data suggest that β-toxin is decreasing host cell survival in two different ways. SMase activity may cause cell death through its production of ceramide, which alone is capable of causing cytotoxicity. However, mutants deficient in SMase activity, and thus not producing ceramide, result in the greatest toxicity to HAECs. The mechanism underlying this toxicity is not known.

Previous research has shown that β-toxin is not cytotoxic to endothelial cells, but these studies were done using HUVECs and at lower concentrations of β-toxin (21). Endothelial cells from different body sites may vary, and this could account for the differences seen. Additionally, we used higher, but likely relevant concentrations of β-toxin to treat cells. β-Toxin production in biofilms, which vegetations resemble, can be ≥15,000 µg/ml; what the production is *in vivo* remains unknown.

Because altered inflammation is thought to be important for the progression of IE and vegetation formation, we examined how β-toxin affects HAEC IL-8 production. We observed that wild-type β-toxin inhibits production of IL-8. This is consistent with previous work by Tajima et al., showing that β-toxin inhibits IL-8 production by HUVECs (21). Treatment with the H289N mutant lacking SMase activity, resulting in overexpression of IL-8, suggests that SMase activity contributes to the ability of β-toxin to inhibit IL-8 production. Accordingly, treatment with ceramide alone inhibits IL-8 production, further supporting this conclusion. Additionally, treatment with the H289N mutant resulted in increased cytotoxicity with increasing protein concentrations; this would lead to a similar stimulation of proinflammatory cytokines. These data support the cytotoxicity findings, suggesting that SMase functions locally to decrease inflammation and thus inhibit resolution of infections by the host, resulting in increased lethality. Surprisingly, our data also showed upregulation of IL-8 production after treatment with biofilm ligase mutants. These data suggest that the biofilm ligase activity of β-toxin is also inhibiting IL-8 production. Thus, the two activities of β-toxin are likely inhibiting cytokine release via two different mechanisms. Another possibility is that the biofilm ligase activity of β-toxin targets the SMase binding site to sphingomyelin on the HAEC membranes. These sites would thus be important for increasing cell interactions with the toxin so that SMase causes its effects.

Inflammation levels during infections are regulated not only by cytokines released from host cells but also by expression of various host cell molecules. We examined how β-toxin affects expression of host cell markers CD40, VCAM-1, and ICAM-1 on HAECs. CD40 is an important transmembrane signaling protein expressed on the surfaces of endothelial cells, whose ligation leads to the production of proinflammatory cytokines. VCAM-1 is a type I membrane protein that mediates cell adhesion and signal transduction. ICAM-1 is an endothelium- or leukocyte-associated transmembrane protein known for its importance in stabilizing cell-cell interactions and facilitating leukocyte endothelial transmigration. We determined that β-toxin and both of its mutant types are capable of upregulating expression of CD40 and VCAM-1, but not ICAM-1, on HAECs.

It is well-known that β-toxin lyses red blood cells by digesting sphingomyelin. How β-toxin affects the overall membrane structure of HAECs, with much lower levels of sphingomyelin in their membranes, has not been examined previously. We observed that HAECs treated with wild-type β-toxin no longer had their typical cobblestone-shaped appearance but were instead rounded. This was also true of cells treated with both mutant types. These data imply that both known activities have effects on membrane integrity. To examine the effects further, we examined HAECs treated with β-toxin stained with antibodies targeting HAEC membranes by immunofluorescence microscopy. We observed that HAECs treated with wild-type β-toxin and biofilm ligase mutants had granular membrane appearances internally. This was not visible in the HAECs treated with the SMase mutant, ceramide, or PBS. Instead they had smooth, evenly stained cell membranes. These data show that the SMase activity of β-toxin disrupts cell membranes even in cell types with lower sphingomyelin content than red blood cells. Previous work has shown that ceramide itself may disrupt endothelial cell membranes, but the disruption of membranes we saw is not due to the production of ceramide through the digestion of sphingomyelin, because treatment with ceramide did not recapitulate the granular appearance. It is likely that these granules are produced from SMase-induced endocytosis of cell membranes. β-Toxin-mediated endocytosis may be a mechanism by which *S. aureus* promotes internalization of virulence factors to cause host cell damage needed in IE pathogenesis. Previous work has shown that a different bacterial SMase causes ATP-independent cell membrane endocytosis, resulting in vesicle formation, but these studies were done in macrophages, fibroblasts, and CHO cells after treatment for no longer than 140 min (22). Furthermore, the investigators observed interactions of SMase-induced vesicles with previously formed endosomes and lysosomes, accounting for the majority of granules (22). Our fluorescence microscopy data with LAMP-1 shows little colocalization of WGA-staining granules with lysosomes, suggesting that there is minimal, if any, interaction in HAECs. Additionally, the previous research observed significant localization of vesicles with Golgi apparatus (22). Our studies indicate no difference in GS15 colocalization with WGA staining in PBS-treated cells compared to β-toxin-treated cells, suggesting that β-toxin is not affecting Golgi interactions in HAECs.

Our studies show that the biofilm ligase activity of β-toxin is important in inhibiting IL-8, CD40 and VCAM-1 expression. We thus propose that the role for this active site is not just to aid in the formation of biofilms. It has previously been hypothesized that the biofilm ligase active site may have many different targets (not just DNA), such as hyaluronic acid, collagen, other proteins, and integral membrane proteins in plasma membranes (18). The binding of the biofilm ligase active site to cell receptors or membrane proteins may be responsible for the unexpected effects we are seeing with mutants inactivated at this site. The contact that the active site has with HAECs could improve interactions of β-toxin by increasing its proximity or stability during association with cells, so that its effects are more pronounced. Our work and previous data show that β-toxin inhibits IL-8 production from endothelial cells (21). Via studies on mRNA levels, research with HUVECs demonstrated that β-toxin treatment reduces transcription of IL-8 (21). Furthermore, these studies also showed that β-toxin blocks phosphorylation of extracellular signal-regulated kinase 1 or 2 (ERK1/2) as a potential mechanism for the reduced transcription (21). It is possible that β-toxin inhibits the phosphorylation of ERK1/2 by binding to or blocking epidermal growth factor receptor by its biofilm ligase site, which could subsequently disrupt downstream signaling cascades and result in the decrease of IL-8 transcription. This would also account for the total kinase levels found with or without treatment remaining the same, since β-toxin would not be targeting the kinases directly (21). This could also explain our IL-8 results with the biofilm ligase mutants at the higher concentrations tested. At these concentrations, the lack of interactions important for inhibiting IL-8 production via binding through the biofilm ligase site are more pronounced such that without these interactions, even though ceramide is being produced, IL-8 production is stimulated.

Since platelet recruitment and activation are critical for the development of IE, to begin to assess how β-toxin may be affecting platelets, we measured platelet aggregation, which is a routine test to assess platelet activation responses. We observed that treating washed platelets with purified β-toxin resulted in strong aggregation responses. Our findings also confirmed that the SMase activity of β-toxin was responsible for platelet activation, as treatment with the mutant lacking SMase activity, but not biofilm ligase activity, resulted in complete loss of the aggregation response. We also used EGTA, a specific calcium chelator, to establish a connection between platelet-platelet interactions that results in the formation of platelet aggregates. Our data clearly demonstrate that β-toxin induces platelet aggregation via integrin-dependent activation, but not due to nonspecific physical agglutination of platelets.

Platelet activation by β-toxin may serve to increase clotting and vegetation formation, which would aid in colonization as well as potentially hide *S. aureus* from the host immune system. Additionally, since bacteremia is a key component in the initiation of IE, it is possible that β-toxin may be present systemically in the host, contributing to the disseminated intravascular coagulation (DIC) that is often seen. This would correspond with our prior *in vivo* data in which β-toxin increases sepsis lethality (20).

Collectively, our data suggest important roles for β-toxin in inflammatory processes at the vascular endothelium, as well as platelet activation during staphylococcal infection. Depending on the concentration of β-toxin present, the toxin is capable of inhibiting or promoting inflammation. This concentration-dependent capacity to regulate inflammation may be important for different types of infections and during different stages of infection. During the initial stages of infection, such as in IE, the toxin may be produced at low concentrations, therefore inhibiting the production of IL-8 and VCAM-1, to delay activation of the host immune system until colonization is established. With colonization, the bacterial load would grow, and therefore, the amount of β-toxin would increase. At these higher concentrations, the protein could cause increased damage by decreasing endothelial cell survival, and it could promote inflammation by inducing expression of proinflammatory cell markers so that vegetation formation propagates. The *S. aureus* USA200 clonal group in particular is adept at causing IE. This clonal group constitutively expresses relatively high levels of β-toxin due to loss of a bacteriophage that typically inactivates the *hlb* gene in other clonal groups. It is likely that this clonal group is significantly associated with causing IE due to its high levels of β-toxin production that would promote a proinflammatory state, leading to vegetation formation. Our previous work with USA400 strains also supports this concept (19). When we introduced a mixed population of USA400 strain MW2 into a rabbit model of IE, a significant proportion of the bacteria we recovered from vegetations had lost the *hlb*-inactivating bacteriophage and thus produced high levels of β-toxin. This indicates that the infection favors inflammation with IE by increasing β-toxin. β-Toxin production may thus be regulated to produce either low or high levels, inhibiting or promoting inflammation, respectively, depending on the infection environment.

## MATERIALS AND METHODS

### Tissue culture.

Immortalized HAECs were prepared and cultured in medium 200 (Gibco Life Technologies, Grand Island, NY) supplemented with low-serum growth supplement (LSGS) and 1% penicillin-streptomycin at 37°C in 5% CO_2_. These cells were previously tested to ensure they retained properties in common with nonimmortalized HAECs. Tissue culture plates (Costar; Corning Life Sciences, Corning, NY) containing 96 wells coated with 1% gelatin were seeded with 7,000 HAECs/ well in 200 µl of medium 200, as determined by hemocytometer counts, and allowed to grow to 80 to 90% confluence. The medium was then removed, and fresh medium 200 containing wild-type β-toxin, SMase mutant (H289N), or a biofilm ligase-deficient mutant (H162A or D163A) at 0.5 µg/ml to 200 µg/ml was added and incubated for up to 24 h. All experiments were performed in triplicate, for a total of nine data points per treatment. Significance was calculated using one-way analysis of variance (ANOVA) analysis.

### Protein production.

Wild-type β-toxin, SMase mutant, and biofilm ligase mutants were cloned into a pTrcHis TOPO vector (Invitrogen Life Technologies, Grand Island, NY) and purified as previously described (20). Following dialysis, proteins were treated with Detoxi-Gel endotoxin-removing resin (Thermo Scientific) to remove possible lipopolysaccharide contamination before concentration through an Amicon Ultra centrifugal filter (Merck Millipore, Germany). Protein concentrations were determined by the Bradford assay kit (Bio-Rad, Hercules, CA) with staphylococcal enterotoxin B as a protein standard. C2 ceramide was purchased from Santa Cruz Biotechnology, Dallas, TX.

### Cytotoxicity and IL-8 enzyme-linked immunosorbent assay.

Following HAEC treatment with β-toxin for 24 h, 100-µl portions of supernatants were removed, and IL-8 was quantified using BD OptEIA human IL-8 enzyme-linked immunosorbent assay (ELISA) set (BD Biosciences, San Diego, CA). HAECs in the remaining 100 µl of medium were coincubated for a 3-(4,5-dimethylthiazol-2-yl)-5-(3-carboxymethoxyphenyl)-2-(4-sulfophenyl)-2H-tetrazolium (MTS) cytotoxicity assay with 20 µl CellTiter 96 AQueous one solution (Promega, Madison, WI) for 4 h. Absorbance was then determined at a wavelength of 490 nm in a Tecan Infinite 200 PRO microplate reader as a measure of cytotoxicity.

### In-cell Western analysis.

Following HAEC treatment with either 25 or 100 µg/ml of β-toxin or 1 µM or 10 µM concentration of ceramide for 24 h, spent medium was removed, and cells were fixed with 50 µl of 3.7% formaldehyde in PBS for 5 min. Formaldehyde was removed, and cells were washed by adding 200 µl PBS for 5 min twice. Cells were permeabilized with 50 µl of PBS containing 0.2% Tween 20 for 5 min and blocked with 100 µl Odyssey blocking buffer (LI-COR, Lincoln, NE) for 1.5 h at room temperature. After the cells were blocked, they were incubated with 50 µl primary antibody diluted in Odyssey blocking buffer overnight at 4°C. Primary antibodies were diluted as follows: (i) CD40 purified mouse anti-human clone 5C3a diluted 1:50 (BD Biosciences, San Diego, CA), (ii) VCAM-1 anti-human antibody diluted 1:200 (Neo Scientific, Cambridge, MA), and (iii) ICAM-1 anti-human antibody (Neo Scientific, Cambridge, MA) diluted 1:200. A solution of PBS containing 0.1% Tween 20 was then used to wash the plates five times. IRDye 800CW infrared dye was used as a secondary antibody with 50 µl of a 1:200 dilution in Odyssey blocking buffer plus 0.2% Tween 20 and incubated for 1 h in the dark. Another wash was completed before adding 50 µl of DRAQ5 (deep red anthraquinone 5) (BD Biosciences, San Diego, CA) diluted 1:10,000 in Odyssey blocking buffer plus 0.2% Tween 20 for 15 min also in the dark. The cells were washed five more times before being allowed to dry in the dark. The LI-COR Odyssey infrared imaging system (LI-COR Biosciences, Lincoln, NE) was used to view the cells, and fluorescence intensity was measured and analyzed with NIH ImageJ.

### Microscopy.

Images were taken at 24 h using a Nikon SMZ 1500 at a magnification of ×70 with a Nikon DS-Ri2 camera and accompanying NIS Elements imaging software version 4.40 (Nikon Instruments Inc., Melville, NY).

For immunofluorescence microscopy, cells were cultured on glass coverslips coated with 1% gelatin in 12-well tissue culture plates (Costar; Corning Life Sciences, Corning, NY) seeded with 50,000 HAECs/well in 1 ml of medium 200 per well, and allowed to grow to 80 to 90% confluence. The medium was then removed, and fresh medium containing 25 µg/ml β-toxin or 5 µM ceramide was added and incubated for 24 h. The spent medium was removed, and the cells were fixed with 1 ml of 2% formaldehyde for 5 min; the cells were washed twice with PBS before staining with 5 µg/ml of wheat germ agglutinin (WGA) Alexa Fluor 488 conjugate (Invitrogen Molecular Probes, Eugene, OR) for 30 min. For Golgi apparatus or lysosome staining, the cells were permeabilized with 0.2% Tween 20 following fixation and blocked with 10% goat serum in PBS containing 1% bovine serum albumin (BSA) plus 0.05% Tween 20 (staining buffer) for 1 h. The cells were then stained with 1:100 purified mouse anti-GS15 antibody (BD Biosciences, San Diego, CA) or 1:20 H4A3 mouse anti-LAMP-1 antibody (deposited in the Developmental Studies Hybridoma Bank [DSHB] by J. T. August and J. E. K. Hildreth) in staining buffer overnight at 4°C. The coverslips were then washed five times for 5 min each time with staining buffer. Secondary goat anti-mouse Alexa Fluor 647 antibody (Thermo Scientific) was then added at 1:200 in staining buffer and incubated for 1 h; WGA staining was then completed as described above. The slides were washed twice with PBS and twice with double distilled water for 2 min between each wash. The slides were then mounted using ProLong diamond antifade mountant with DAPI (4′,6′-diamidino-2-phenylindole) (Molecular Probes, Eugene, OR), and images were captured using a fluorescence microscope (Olympus BX61; Olympus) with an Olympus DP73 camera and accompanying OlyVIA 2.9 imaging software.

### Platelet aggregation assays.

Tyrode’s gelatin solution without calcium (TG-no Ca) and Tris-Tyrode’s gelatin solution (TTG) were prepared by the method of Noguchi et al. (23). Platelets were isolated by differential centrifugation from fresh rabbit blood. Briefly, blood was collected from an adult New Zealand White rabbit by cardiac puncture into citrate-phosphate-dextrose (7:1, vol/vol of blood:citrate-phosphate-dextrose solution) (26.3 g trisodium citrate, 3.27 g citric acid, 25.5 g dextrose, 2.22 g monobasic sodium phosphate per liter, pH 5.6). Platelet-rich plasma (PRP) was obtained by centrifugation of blood at 130* *×* * g for 15 min. PRP was mixed with 1 µM prostaglandin E1 (PGE1) and centrifuged at 800* *×* * g for 10 min, and then sedimented platelet pellets were washed with TG-no Ca buffer containing 1 µM PGE1. Platelets were finally resuspended in TTG buffer to a final concentration of 4 × 10^8^ platelets/ml. In order to study platelet aggregation, washed platelets were stirred at 1,200 rpm at 37°C in a cuvette of an aggregometer (Chrono-Log model 560-VS) for 1 min, after which either collagen (2 µg/ml) (a positive control) or β-toxin (2.5 µg/ml) was added to the cuvette, and light transmittance was recorded. Aggregation was measured as percent change in light transmission, where 100% refers to transmittance through a blank solution. Aggregation of washed platelets was confirmed with 2 µg of collagen as a control prior to each experiment. For experiments involving EGTA, a final concentration of 5 mM EGTA was added to the washed platelets 2 min prior to the addition of the β-toxin/collagen.
